# The role of dorsal premotor cortex in joint action inhibition

**DOI:** 10.1038/s41598-024-54448-4

**Published:** 2024-02-26

**Authors:** Elisa Dolfini, Pasquale Cardellicchio, Luciano Fadiga, Alessandro D’Ausilio

**Affiliations:** 1 IIT@UniFe Center for Translational Neurophysiology, Istituto Italiano di Tecnologia, Via Fossato di Mortara, 17-19, 44121 Ferrara, Italy; 2 Department of Neurosciences and Rehabilitation Section of Physiology, Università di Ferrara, Via Fossato di Mortara, 17-19, 44121 Ferrara, Italy; 3 Physical Medicine and Rehabilitation Unit, IRCCS Istituto Giannina Gaslini, Genoa, Italy

**Keywords:** Joint action, Inhibition, TMS, Silent period, GABA, Dorsal and ventral premotor areas, Neuroscience, Physiology

## Abstract

Behavioral interpersonal coordination requires smooth negotiation of actions in time and space (joint action—JA). Inhibitory control may play a role in fine-tuning appropriate coordinative responses. To date, little research has been conducted on motor inhibition during JA and on the modulatory influence that premotor areas might exert on inhibitory control. Here, we used an interactive task in which subjects were required to reach and open a bottle using one hand. The bottle was held and stabilized by a co-actor (JA) or by a mechanical holder (vice clamp, no-JA). We recorded two TMS-based indices of inhibition (short-interval intracortical inhibition—sICI; cortical silent period—cSP) during the reaching phase of the task. These reflect fast intracortical (GABAa-mediated) and slow corticospinal (GABAb-mediated) inhibition. Offline continuous theta burst stimulation (cTBS) was used to interfere with dorsal premotor cortex (PMd), ventral premotor cortex (PMv), and control site (vertex) before the execution of the task. Our results confirm a dissociation between fast and slow inhibition during JA coordination and provide evidence that premotor areas drive only slow inhibitory mechanisms, which in turn may reflect behavioral co-adaptation between trials. Exploratory analyses further suggest that PMd, more than PMv, is the key source of modulatory drive sculpting movements, according to the socio-interactive context.

## Introduction

During multi-agent action coordination (or JA) a continuous and reciprocal exchange of movement-related information is essential to achieve a shared goal^1–4^. Partners in a JA have to integrate local information about others’ action (i.e., what my partner is doing and how) as well as information regarding the history of interaction (i.e., what my partner did in previous trials) in order to appropriately steer one’s own motor output. This process of integration necessarily results in local motor adjustments (i.e., online motor corrections) and long-term adaptations (i.e., fine-tuning/changing of movement plans between trials). The regulation of these motor processes on different scales is probably based on inhibitory mechanisms^5^. Indeed, volitional motor control relies on the balance between mechanisms for initiating movements and those for suppressing undesired movements^6^. Recent evidence suggests that intracortical and corticospinal inhibitory mechanisms may be regulated to enable the dynamic motor exchanges that characterize JA tasks^7^. To date, however, very little is known about the cortical areas that contribute to the modulation of motor inhibition, during JA.

The lateral premotor cortex, which provides an important modulatory drive to corticospinal projections^8–11^, is a likely candidate. Premotor areas are essential for planning, preparing and monitoring reaching and grasping movements^12–14^. The ventral sector (PMv) is active during both grasping action execution and observation^15^ and is a key component of the brain-wide network recruited for others’ action goal anticipation^16^. Cortico-cortical circuits connecting the PMv and the primary motor cortex (M1) are modulated during the observation of other’s grasping action^17^ and perturbation of PMv modulates corticospinal excitability during hand action observation^18^. The dorsal sector (PMd) is classically regarded as a motor preparation area for reaching^19^, encodes the spatial coordination of whole arm movements and is also recruited during similar action observation^20,21^, thus potentially playing an important role in JA motor co-regulation^22^. In fact, a class of neurons in the monkey PMd encode movement properties in a joint isometric center-out task^23^. However, little is known about the differential role played by the dorsal and ventral premotor areas in guiding human JA, and specifically in modulating JA motor inhibition.

**Figure 1:**
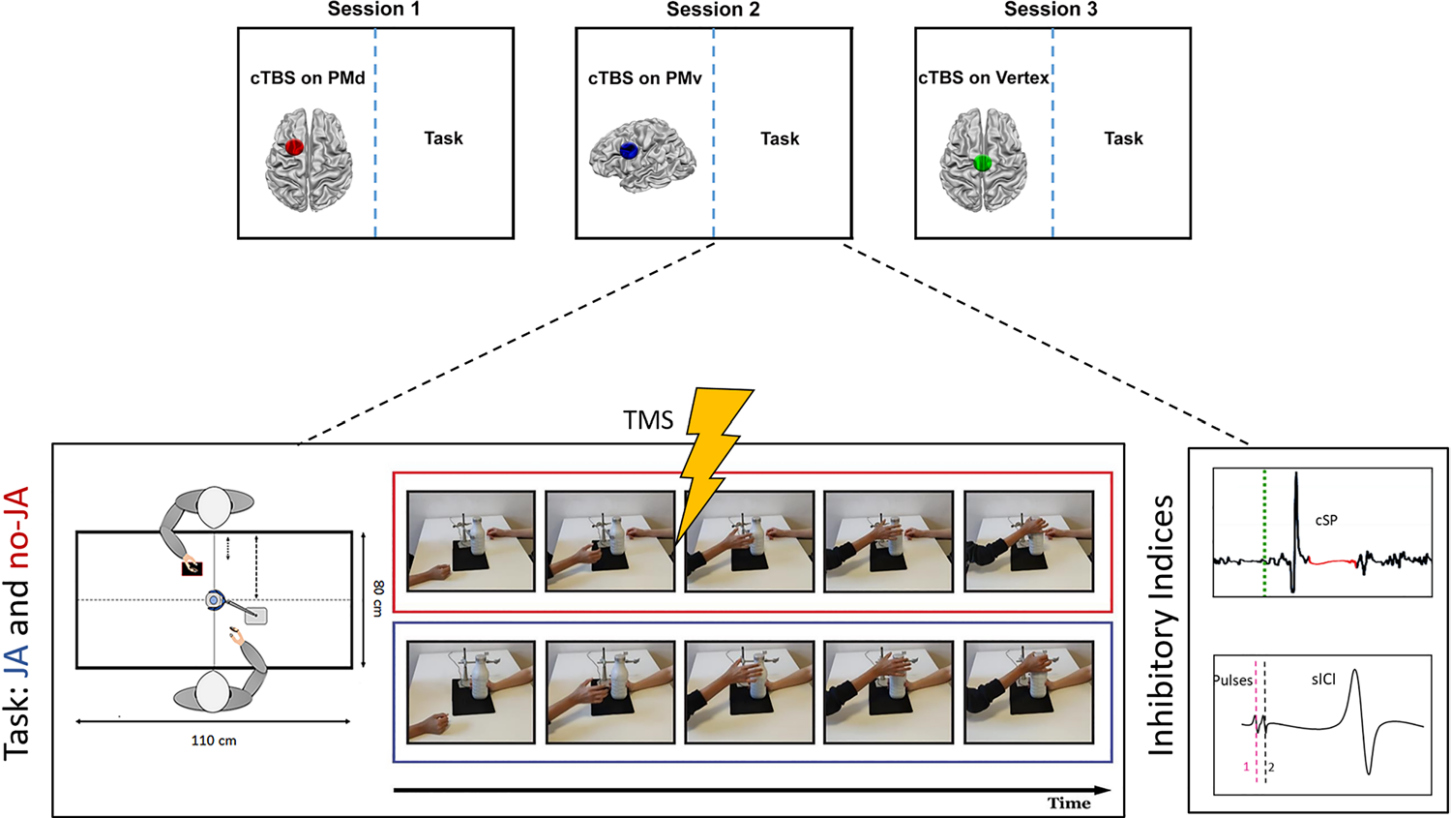
The figure shows the TMS stimulation sites, the set-up and the actions performed by the participants. Above, the three experimental sessions are indicated. In every session the cTBS was used on one of the target: the vertex in green, the PMd in red, the PMv in blue. The order of the sessions was randomized across participants. Below, the task is explained. The Red box indicates the no-JA condition, the blue box indicates the JA one. In both conditions, after the go-signal participants were instructed to reach for the bottle and open the cap by unscrewing it. The bottle was held by a co-actor or by a mechanical holder. In the no-JA condition the actor was seated in the same position but kept her hand resting on the table. In both conditions the mechanical holder and the co-actor were constantly present and visible to participants.

In the present study, we used a JA task that was developed and validated by our group^7^ (Fig. 1; a variation is present also in another work^24^). Participants are asked to reach for a bottle and open it with one hand by unscrewing the cap. The key manipulation is that the bottle is held stable either by a co-actor (JA) or a mechanical holder (vice clamp, no-JA). This task, being a bimanual action transformed into a unimanual dyadic task, forces one key aspect of JA: one participant alone cannot achieve the goal without the cooperation of another^7^. In fact, the confederate holding the bottle (JA only) increases the grip force while the participant is still moving towards the bottle until reaching a plateau only later, during the haptic exchange necessary to open the bottle. Considering that the squeezing force changes during the reaching phase, the participant has to apply fine and fast online adjustment of the reaching plan, on the fly. As a consequence, the task is geared towards the study of mutual and dynamic co-adaptation between partners during joint action.

In a previous study (by Cardellicchio et al.^7^), by means of Transcranial Magnetic Stimulation (TMS), we measured (i) corticospinal excitability (CSE), (ii) cortical silent period (cSP) and (iii) short-interval intracortical inhibition (sICI), during the reaching phase of the task. The CSE, measured with a single pulse TMS protocol, provides a combined readout of the strength of the synaptic input converging on pyramidal projection neurons and spinal excitability^25^. As a consequence, CSE reflects the state of excitation of the corticospinal system as a whole. The cSP is only visible during active muscle contraction and consists of a period of muscular inactivity following the Motor Evoked Potential (MEP) elicited by single TMS pulse. The cSP reflects the magnitude of slow corticospinal (GABAb-mediated) inhibition^26^. Conversely, sICI reflects fast intracortical (GABAa-mediated) inhibition^27^ which is instead measured with a paired-pulse TMS protocol^28^. In our previous study, we found no modulation of CSE, while we found an increase of slow corticospinal inhibition (cSP) and a reduction of fast intracortical inhibition (sICI) in JA with respect to no-JA. Therefore, the two inhibitory indexes were modulated in the JA versus no-JA comparison, but in the opposite direction, thus suggesting a dissociable role of the two inhibitory mechanisms^7^. In light of these results, we concluded that the fine regulation of fast and slow neurophysiological inhibition might be essential in our JA task^29^.

In this new research, in a three sessions experiment, we applied an offline repetitive TMS stimulation (cTBS) to the two premotor areas (lPMd or lPMv) or on the vertex (as control site; Fig. 1). In each session, during both the JA and the no-JA tasks we measured, exactly as in our previous study, (i) CSE, (ii) cSP (iii) sICI. The rationale of this study is to directly test whether we can replicate the same neurophysiological pattern of results in the control condition (vertex) and whether manipulating the activity of premotor areas (dorsal or ventral) induces specific modulation of these inhibitory indexes.

Therefore, when applying cTBS to the vertex, we expect to replicate the reduction of sICI and an increase for the cSP in JA (when contrasted with no-JA). Moreover, considering that cSP modulation was shown to reflect the history of trial-by-trial interaction between partners^7^, we predict premotor activities would be essential in keeping track of these adaptations across trials. Instead, we predict no cTBS-induced modulations for sICI, because fast intracortical inhibition might reflect only local motor adaptations that do not transfer form trial to trial. Furthermore, given the inherent motor nature of our task, we predict also a larger contribution from the PMd. In fact, PMd might be essential in tasks requiring mutual motor adaptations^22^ while PMv might instead code higher-order joint action goals^30^.

## Materials and methods

### Subjects

Fifteen healthy naive volunteers took part in the study (males: 7; mean age: 24.7, SD ± 2.9), and all of them participated at the three experimental sessions. The sample size was defined with a power analysis based on the main results of our previous experiment^7^, specifically the t-test on cSP data. The power analyses indicated that a sample size of 15 participants was necessary to achieve a statistical power (1 − β) of 0.80 (α = 0.05; effect size f = 0.36^7^; number of measurements = 3; correlation = 0.5, analysis performed with G ∗ Power 3.1.9.7 software^31^). All participants took part in the three experimental sessions. Subjects were right-handed, as assessed by the Edinburgh Handedness Inventory^32^. They were informed about the experimental procedure and gave their written consent according to the 1964 Helsinki Declaration, as revised in 2013. None of the participants reported neurological, psychiatric or other contraindications to TMS^33^. The experiment was approved by the ethical committee “Comitato Etico Unico della Provincia di Ferrara” (approval N. 170,592), and participants were compensated for their participation with 60 € (in total for the three sessions).

### Task, stimuli and procedures

After being informed about the experimental procedure and having signed the informed consent, participants were submitted to a TMS mapping procedure, motor threshold (MT) assessment and cTBS administration (see next section for the details about these procedures). In three separate experimental sessions, we applied cTBS to lPMd, lPMv, or the vertex as a control. A minimum of five days elapsed between each session and their order was randomized across participants. After cTBS, participants performed the behavioral task while single pulse TMS (SP-TMS) and paired pulses (PP-TMS) protocols were applied (see TMS and EMG paragraph) to assess the variables of interest (CSE, sICI and cSP).

During our task, participants had to open, with their right hand, a bottle that needed to be stabilized. The bottle was held by an actor (Joint Action condition; JA) or by a mechanical holder (no Joint Action—no-JA; Fig. 1), but the actor remained seated in front of the subject in both conditions (as depicted in Fig. 1 and fully described in^7^.

Participants were seated in a comfortable armchair with their right forearm pronated and the hand resting on a button-box (cedrus RB-840) positioned on a table in front of them (table: length = 110 cm, width = 80 cm). A semi-deformable plastic bottle was positioned on the table at a distance of approximately 2/3 of the participant’s arm length from the participants’ chest along their midline. The plastic bottle had a rough texture, measured 25 cm, and its cap was 5 cm in diameter. All the participants started the trial from the same initial position (pressing a button on the table). Each trial began with the presentation of a 300 ms sine-wave tone (800 Hz), instructing participants to reach and open the screw cap. The participants were asked to reach and unscrew the cap and, once completed, move back to the original position by pressing the start button. The cap contained a capacitive sensor to detect the instant of touch. Reaching onset was defined by button release, reaching offset by the touch of the cap. The length of the inter-trial interval (ITI) was 7 s, with a randomized jitter of ± 500 ms.

During the behavioral task, two TMS protocols were adopted in 50% of the trials (see next section for more details): (1) SP-TMS and (2) PP-TMS protocols. Each condition (JA, no-JA) contained 60 trials, of which 15 were stimulated with SP-TMS, 15 with PP-TMS, and 30 without TMS (catch trials). The TMS pulse was delivered during the reaching phase of the movement. In fact, intrinsic hand muscles that are necessary to unscrew the bottle cap are normally active far before grasping. For this reason we triggered the TMS when the level of OP muscle contraction exceeded a threshold (using the same device and criterion used in our previous study and described later). In this way, all indices were recorded when the exact same level of muscle contraction was present. The entire experimental session contained 120 trials separated into four blocks (two blocks for JA and two for no-JA, each with 30 trials) in a counterbalanced order across participants. Additionally, at the beginning and end of the experiment, we also collected 15 SP-TMS and 15 PP-TMS to verify that our neurophysiological indices did not change across the experimental session. The task was approximately 25 min long per participant, while the whole experimental session lasted about 75 min (including the informed consent, neuronavigation, TMS mapping, threshold estimation and cTBS administration). The experimental control of the events was implemented in MATLAB (MATLAB R2015b, MathWorks Inc., Natick, MA, 2000).

### TMS and EMG

During the mapping session, we assessed the active and resting motor threshold (respectively aMT and rMT) for the right Opponens Pollicis (OP) muscle. The aMT was defined as the lowest TMS intensity that elicits a motor evoked potential (MEP > 100 μV) when participants maintained a slight contraction of the right OP (∼ 10% of the maximum voluntary contraction) in at least 5 of 10 consecutive trials^34^. The aMT was assessed by a handheld figure-of-eight coil (70 mm air film coil external diameter at each wing; Magstim Co., Ltd.) connected to a Magstim biphasic stimulator (Magstim Super Rapid2 Plus1 unit, Whitland, UK). The mean (± SEM) aMT across the participants was 50 ± 6.41% of the maximum stimulator output. Coil and stimulator were the same as those employed to administer the cTBS protocols.

The rMT was established as the lowest stimulus intensity eliciting a MEP on the target muscle (MEP > 50 μV). The rMT was assessed by a handheld figure-of-eight coil (70 mm external diameter at each wing; Magstim Co., Ltd.) connected to a Magstim monophasic stimulator (Magstim 200 BiStim units, Whitland, UK). The mean (± SEM) rMT across the participants was 42.5 ± 5.2% of the maximum stimulator output. Coil and stimulator were the same as those employed in the SP-TMS and PP-TMS protocols.

In both cases, the TMS coil was held tangentially to the scalp, with the handle pointing backward and laterally to form a 45° angle with the midline. Optimal OP localization was marked on an elastic cap. The OP was chosen because it is critical in grasping and rotating movements required to open a screw cap. The EMG signal was recorded using a wireless EMG system (Zerowire EMG, Aurion, Italy) with pairs of Ag/AgCl surface-adhesive electrodes (5 mm in diameter) placed with a tendon-belly montage. EMG data were digitized (2 kHz) and acquired using a CED power3A 1401 board to be visualized on a monitor’s PC (Signal 6.09 software; Cambridge Electronic Design, Cambridge, UK).

The PMd stimulation site was localized in relation to the motor hot spot, 2.5 cm anterior and 1 cm medial, as recommended in previous reports^35,36^. The PMv location (MNI coordinates: − 52.8, 11.6, 25.1) was estimated by neuronavigating (SofTaxic, E.M.S., Bologna, Italy) on a MNI template brain using digitized skull landmarks (nasion, inion, and preauricular points) and 23 scalp points provided by a Polaris Vicra optical tracker (Northern Digital, Canada). The vertex, as control site, was chosen because it is quite unlikely to affect other potentially task-relevant brain areas (i.e., supplementary motor area—SMA)^37^. In fact, the stimulation of SMA requires the coil to be placed 4 cm anterior to the Cz position and higher stimulations to reach deeper within the longitudinal fissure. Although the perfect control does not exists, a real stimulation of the vertex produces a far more realistic sensation than other strategies often employed to induce sham stimulations (i.e., rotated coil, foam or wooden slabs).

We used a cTBS protocol to produce a lasting suppression of regional excitability in the stimulated cortex^38^. The cTBS protocol consists of the repeated administration of short high-frequency bursts. Each burst consists of three pulses with an inter-stimulus interval (ISI) of 20 ms (50 Hz). The high-frequency triple-pulse bursts are repeated every 200 ms (5 Hz). The 600 pulses cTBS protocol therefore lasts 40 s. Pulse intensity was defined on an individual basis as 80% of the aMT^39,40^. During the cTBS on PMd and PMv the coil was oriented at a 45° angle to the midline with the handle pointing backwards. At the control cTBS site (vertex), the coil handle was oriented parallel to the longitudinal fissure and pointing backward.

After cTBS, the participants rested for 5 min without moving their hands or feet^41,42^. Based on previous findings^38,43^, the time window of reduced excitability following cTBS is expected to last between 20 and 30 min. During cTBS, EMG activity was monitored to exclude stimulation of the ipsilateral M1. The absence of any current spread toward the motor cortex was confirmed by the lack of motor-evoked responses.

During the task, SP-TMS protocols were used to measure corticospinal excitability (CSE) and cortical silent periods (cSP), whereas PP-TMS was used to measure short intracortical inhibition (sICI). SP-TMS was delivered at 120% of rMT. For PP-TMS, the conditioning stimulus (CS) was set at 80% of rMT, while the test stimuli (TS) were set at 120%, with an inter-stimulus interval (ISI) of 3 ms. The coil, placed on the OP muscle scalp location, was oriented at a 45° angle to the midline with the handle pointing backwards inducing a postero-anterior current flow^44,45^.

The experiment is an exact replication of the task we used in our previous experiment^7^. As in our previous experiment, TMS (SP-TMS or PP-TMS) was triggered, in each trial, based on OP muscle activity during the reaching phase. Such an EMG-based triggering of TMS ensures that neurophysiological indexes are locked to a functionally relevant event as opposed to rely on an arbitrary and fixed time point. Specifically, a moving average procedure with a sliding window of 50 ms was run online during reaching actions on the rectified surface EMG. The OP onset was defined as the instant at which the EMG signal exceeded 100% of both the average EMG recorded in a 100 ms window before reaching started and the EMG signal in the preliminary part of reaching phase. TMS pulses were delivered 100 ms after the OP onset.

### Analysis

MEP size was extracted by computing peak-to-peak amplitudes in a window of 60 ms following the TMS pulse. CSE was then normalized as the ratio between the mean MEP size within each condition and baseline mean MEP size (baseline data collected before and after the experiment and thus at rest). The sICI values were expressed as the ratio between the mean conditioned MEP amplitude and mean single-pulse MEP amplitude. Silent period durations were measured for each trial as the time between the offset of the MEPs and the return of EMG activity according to standard procedures^46–49^. The end of the cSP was determined for each individual trial as the resumption of at least 2 SD of EMG activity to the level of the pre-TMS stimulus (end of cSP > 2SD of the 50 ms pre-stimulus signal). Offline semi-automated extraction of MEPs amplitudes and cSP durations was performed using Signal 6.05 software (Cambridge Electronic Design, Cambridge, UK). We discarded from the sICI (6.2%—analyzed trials: 84.4 ± 7.4) and CSE (5.9%—analyzed trials: 84.6 ± 6.9) analysis all trials with either MEP below 50 µV or trials in which the subjects touched the cap before the TMS pulse. For the cSP analysis, we discarded all trials with no visible cSP and those in which the subjects touched the cap before the TMS pulse (5.8%; mean: 84.9 ± 7).

First, we analyzed the CSE and sICI of the baseline trials (cSP was not measured because participants were at rest) acquired at the beginning and at the end of the experiment. We performed two separated one-way ANOVAs, one for CSE and one for sICI respectively, with SITES (PMv, PMd, Vertex) as factors, to exclude unspecific excitability/inhibition changes due to cTBS stimulation.

We then compared JA and no-JA conditions for each neurophysiological index (CSE, cSP, and sICI) in three 3X2 repeated measures ANOVAs with factors SITES (PMv, PMd, Vertex) and CONDITIONS (JA, no-JA). The main target of this analysis was to replicate the dissociation between the two inhibitory indexes on the vertex, and observe if they were modulated after cTBS stimulation on premotor sites. Significant interactions emerging in this analysis step motivated subsequent specific comparison. In fact, for cSP only, we computed the ratio between JA and no-JA values to test the net contribution of different stimulation sites to the modulation of this index. The ratio values were analyzed using a one-way ANOVA with factors SITES (PMv, PMd, Vertex). Significant main effects or interactions were explored via Newman-Keuls post hoc analyses. All analyses were performed using STATISTICA 9 software (StatSoft, Inc.).

We also run a Bayesian repeated measure ANOVA according to the ANOVA designs described before, on sICI and cSP, then followed by Bayesian t-tests to perform specific comparisons. These analyses were conducted to further confirm the strength of the evidences in favor of the presence of an effect on cSP and the absence of an effect on sICI. Indeed, Bayesian analyses are better suited to differentiate between “absence of evidence” and “evidence of absence”^50^. Bayesian analyses were performed using JASP v0.17.1 software (Jeffreys’s Amazing Statistics Program, JASP team, 2019).

### Results

Preliminary analyses focused on assessing the influence of cTBS (all three sites) on neurophysiological indices at rest. Analysis of baseline CSE and sICI confirm their stability across the three stimulation sites (CSE: F (2,28) = 0. 17; *p* = 0.84; η2 = 0.01; sICI: F (2,28) = 0.77; *p* = 0.47; η2 = 0.05). This result shows that there were no non-specific baseline changes between recording sessions.

### Results on CSE

We then moved to the main analyses that were aimed at searching for specific task (JA and no-JA) and site (PMv, PMd, Vertex) interactions. The 3X2 ANOVA on CSE shows no differences across conditions (JA/no-JA: F (1,14) = 2.66; *p* = 0.12; η2 = 0.16), stimulation sites (F (2,28) = 0.17, *p* = 0.84, η2 = 0.01), nor interaction effects (F (2,28) = 0,06; *p* = 0.93; η2 = 0.004). This result confirms our previous observation^7^ and extend the idea that CSE is not modulated during JA coordination also when cTBS is applied to the premotor cortices.

### Results on sICI

**Figure 2:**
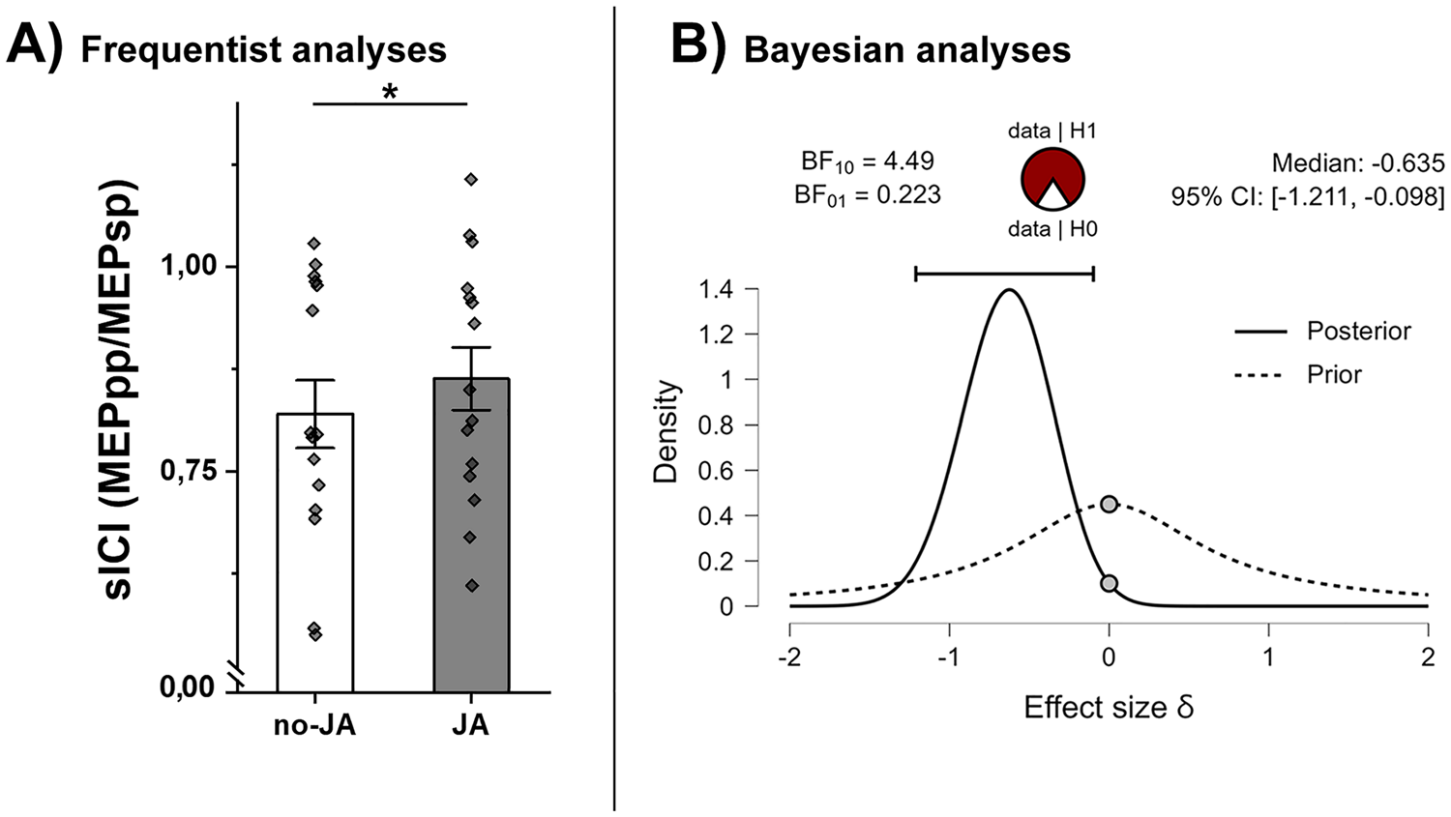
Inhibitory indices: sICI. Panel (**A**) shows frequentist analyses on the modulation of sICI in the no-JA and JA conditions. Asterisks denote significant effects. Panel (**B**) shows Bayesian two-sample t-test for the parameter δ on sICI. The circle at the top indicates the probability of the null (H_0_) and the alternative hypotheses (H_1_). The two gray dots indicate the prior and posterior density at the test value. In the top right corner is shown the median and the 95% central credible interval of the posterior distribution. The Bayesian t-test compares JA vs no-JA. The test indicates a moderate evidence in favor of the H_1_ (BF_+0_ = 4.49).

The 3X2 ANOVA on sICI confirms our previous result^7^, showing a significant reduction of fast intracortical inhibition in the JA condition (as opposed to no-JA). In fact, the ANOVA on sICI shows a main effect of CONDITIONS (F (1,14) = 8.08; *p* < 0.05; η2 = 0.36; JA: mean: 0.86 ± 0.14 SD; no-JA: mean: 0.81 ± 0.15 SD; Fig. 2A), no main effect of SITE (F (2,28) = 0.44; *p* = 0.65; η2 = 0.03), nor the interaction (F (1,14) = 1.2; *p* = 0.31; η2 = 0.07).

As an extension and to confirm the results observed with a frequentist approach we employed a Bayesian 3X2 repeated measure analysis, comparing all models to the model that best predicts the data. The Bayesian ANOVA on sICI reveals that the only factor that explains the data (the best model) is CONDITIONS (JA vs no-JA). The result on SITE reveals moderate evidence for the absence of a modulation (BF10 = 0.15). Also the interaction between SITE and CONDITIONS reveal a similar Bayes factor (BF10 = 0.13) thus supporting the claim that cTBS did not affect sICI, regardless of condition (Table 1). Subsequently, we used post-hoc Bayesian t-tests to obtain Bayesian confidence intervals (CIs) for specific contrasts of interest (JA vs no-JA; Table 2). We find a moderate evidence for larger inhibition in no-JA condition (BF + 0 = 4.49 with median posterior δ = − 0.635; 95% CI = [− 1.211, − 0.098]; see Fig. 2B). Summing up, sICI is reliably modulated by CONDITIONS (JA vs no-JA; in full agreement with our previous study), but stimulation SITE did not affect such a modulation.

**Table 1:** Bayesian repeated measure ANOVA on sICI.

Models	P(M)	P(M|data)	BF_M_	BF_10_	Error %
Condition	0.200	0.470	3.551	1.000	
Site + Condition	0.200	0.223	1.147	0.474	9.853
Null model (incl. subject and random slopes)	0.200	0.170	0.820	0.362	1.469
Site	0.200	0.074	0.319	0.157	8.433
Site + Condition + Site ✻ Condition	0.200	0.063	0.268	0.134	13.021

The table shows the models that could predict data. The first model presented is the best one and the others are compared with it. In our case the best model includes the main effect of Condition (JA vs no-JA). The second and third columns display the prior model probabilities (P(M)) and posterior model probabilities (P(M|data)), respectively. The fourth column (BF10), shows the Bayes Factor relative to the best performing model. Lastly, the final column indicates the relative error associated with the numerical method used to approximate the Bayes factors. Here we can assume that there is moderate evidence for the absence of a Site effect (BF10 = 0.15). Moreover, the same result is observed for the interaction (BF10 = 0.13). Absence of evidence is shown for the null model (BF10 = 0.36) and the simultaneous presence of the two main effect of Site and Condition (BF10 = 0.47).

**Table 2:** Bayesian t-tests on sICI.

Measure 1	Measure 2	BF_10_	Error %
no-JA	JA	4.490	1.221 × 10^–6^

This table reports the Bayes factor for a paired sample t test. A low value of “Error %” indicate greater numerical stability of the result. In this case the alternative hypothesis specifies that Measure 1 is smaller than Measure 2. (e.g., no-JA shows more inhibition than JA).

### Results on cSP

**Figure 3:**
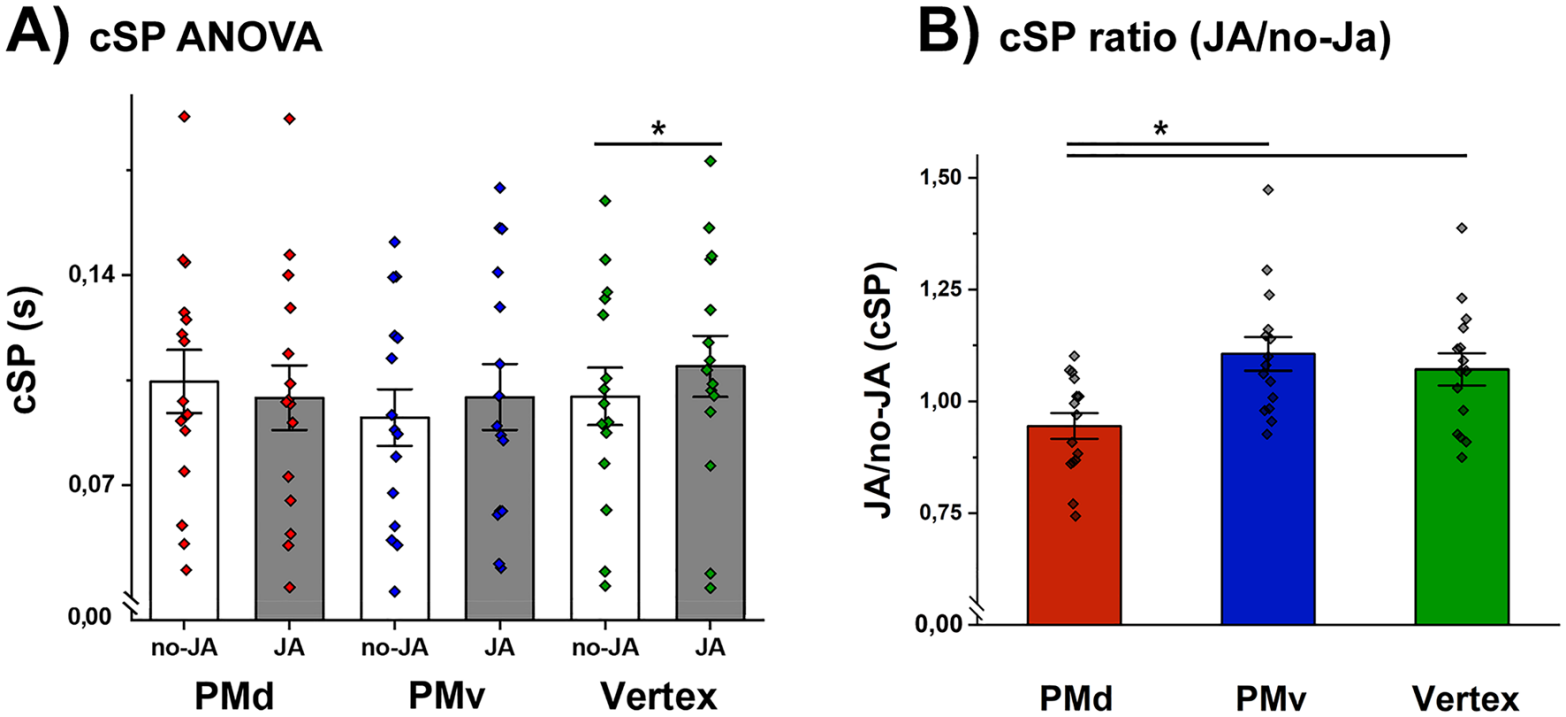
Inhibitory indices: cSP. In panel (**A**) we show that larger inhibition for JA was present only after the stimulation of the Vertex. After cTBS stimulation of the two premotor areas we observe no specific modulation. In panel (**B**) we show the analyses based on the ratio between JA and no-JA for the three different stimulations sites (mean and standard error). Asterisks denote significant effects.

Moving to the analyses of cSP data, the 3X2 ANOVA shows no main effect of SITES (F (2,28) = 1.06, *p* = 0.35, η2 = 0.07) or CONDITIONS (F (1,14) = 4.01; *p* = 0.06; η2 = 0.22), while an interaction between SITES and CONDITIONS (F (2,28) = 5.49, *p* < 0.05; η2 = 0.28) is present. The post-hoc analyses reveal that after the vertex stimulation (the control condition) the cSPs were longer (more inhibition) in the JA condition (JA_(vertex)_ mean:0.109 ± 0.039 SD) than in the no-JA condition (no-JA_(vertex)_ mean:0.099 ± 0.036 SD; *p* = 0.019), replicating the result in our previous experiment^7^. Instead, we do not find modulation between JA and no-JA after both PMv and PMd stimulation. Specifically, no-JA_(PMv)_ (mean:0.092 s ± 0.03 SD) is not different from JA_(PMv)_ (mean: 0.099 s ± 0.04 SD; *p* = 0.42). Similarly, after PMd stimulation no cSP modulation is found (no-JA_(PMd)_: mean: 0.105 ± 0.04 SD; JA_(PMd)_: mean: 0.099 ± 0.04 SD; *p* = 0.14; Fig. 3A).

**Figure 4:**
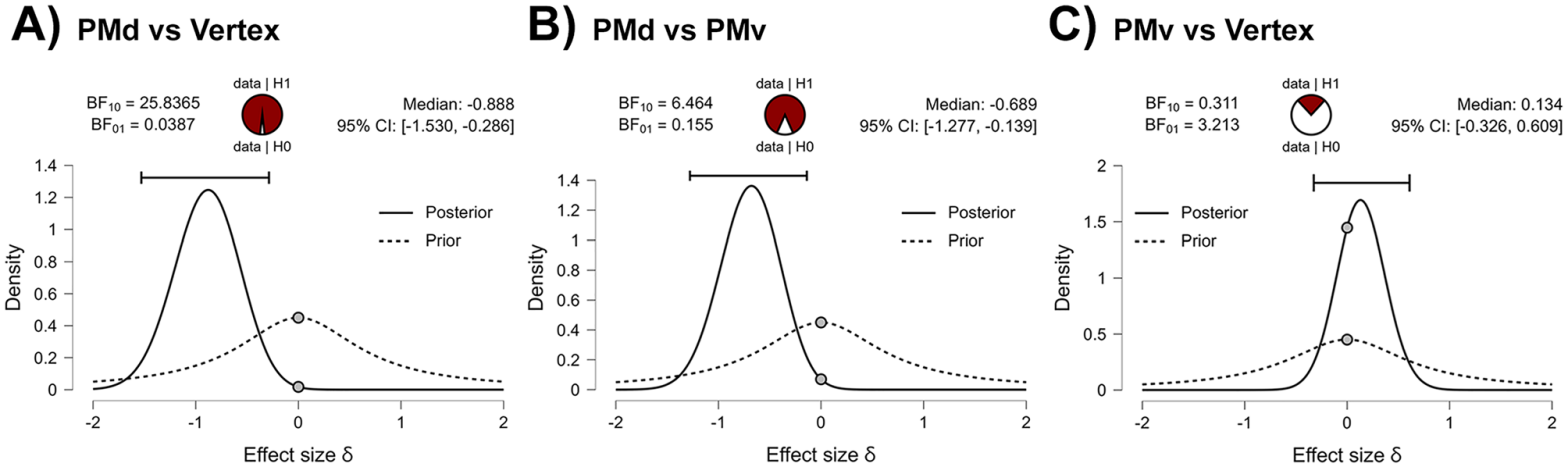
Bayesian two-sample t-test on JA/no-JA ratios for the parameter δ on cSP. The circle at the top indicates the probability of the null (H_0_) and the alternative hypotheses (H_1_). The two gray dots indicate the prior and posterior density at the test value. In the top right corner of each panel are shown the median and the 95% central credible interval of the posterior distribution. Panel (**A**) represent the comparison between the PMd stimulation and vertex stimulation. We observe a strong evidence for H_1_ with a BF_+0_ = 25.837 (PMd smaller than Vertex). In panel (**B**), comparing PMd and PMv, we observe a similar result, with BF_+0_ = 6.464, in favor of the H_1_ (PMd smaller than PMv). The Panel (**C**) shows the absence of evidence for a difference between PMv and Vertex (BF_+0_ = 0.311, in favor of H_0_).

A follow-up ANOVA on JA/no-JA ratios was then employed to increase the sensitivity towards modulation across the two conditions of interest. We show a significant effect of stimulation site (F (2,28) = 6.239; *p* = 0.005; η2 = 0.3; Fig. 3B) and the post-hoc tests further clarify that the cSP ratios in PMd (mean: 0.94 ± 0.11 SD) are smaller than in PMv (mean: 1.07 ± 0.13 SD; p = 0.01) and vertex (mean: 1.1 ± 0.14 SD; *p* = 0.006; Fig. 3). The Bayesian one-way ANOVA on cSP ratios reveal that we have enough evidences to assert that the stimulation SITE is the best model to explain the data (BF_M_ = 20.738; Table 3). The post-hoc Bayesian t-tests show strong evidence for the difference between PMd and Vertex (BF + 0 = 25.84 with median posterior δ = -0.888; 95% CI = [− 1.53, − 0.286]; see Fig. 4A and Table 4) and moderate between PMd and PMv (BF + 0 = 6.46 with median posterior δ = − 0.689; 95% CI = [− 1.277, -0.139]; see Fig. 4B and Table 4). No difference is found between the PMv and Vertex (BF + 0 = 0.311 with median posterior δ = 0.134; 95% CI = [− 0.326, 0.609]; Fig. 4C and Table 4). These results suggest that the JA vs. no-JA pattern of cSP modulation, confirmed in the control stimulation area, is fundamentally altered after PMd stimulation.

**Table 3:** Bayesian one-way ANOVA on cSP ratios (JA/no-JA).

Model comparison on cSP ratio					
Models	P(M)	P(M|data)	BF_M_	BF_10_	Error %
site	0.500	0.954	20.738	1.000	
Null model (incl. subject and random slopes)	0.500	0.046	0.048	0.048	0.744

The table shows that comparisons between stimulation sites is the best model that explain the data (BF_M_ = 20.738).

**Table 4:** Bayesian t-tests on cSP ratios.

Measure 1	Measure 2	BF_10_	Error %
PMd	Vertex	25.837	1.243 × 10^–6^
PMd	PMv	6.464	1.185 × 10^–6^
PMv	Vertex	0.311	0.012

This table reports the Bayes factor for a paired sample t test. A low value of “Error %” indicate greater numerical stability of the result. In this case we observe strong evidence for PMd lower than Vertex (BF_+0_ = 25.837) and a moderate evidence for PMd lower than PMv (BF_+0_ = 6.464).

## Discussion

In this experiment we explored the role of the lateral premotor cortex in modulating indexes of neural inhibition during JA coordination. First of all, in the control condition (vertex stimulation) we showed a dissociation between fast intracortical (sICI) and slow corticospinal inhibition (cSP): sICI was reduced while cSP was increased during the JA as opposed to the no-JA condition. We could thus replicate the dissociation between fast and slow inhibition that was previously linked to the parallel processing of local motor adjustments (i.e., online motor corrections) and long-term adaptations (i.e., fine-tuning/changing of movement plans between trials) during JA^7^.

We now add relevant information on which of these two processes may depend from signals originating in the premotor cortex. Specifically, we observed that sICI modulation was not influenced by cTBS applied on the PMv or PMd. In contrast, the cSP showed its dependence on cTBS applied to both premotor areas. While after PMv stimulation the direction of the modulation remained qualitatively the same (more inhibition in JA, Fig. 3A), cTBS on PMd determined an increase of cSP duration in no-JA trials (more inhibition in no-JA, Fig. 3A). This latter differentiation between PMv and PMd, was more effectively confirmed by Bayesian analyses on JA/no-JA ratios. Considering that PMd is clearly central in the inhibitory control required in motor execution^51,52^, our current study provides evidence that PMd, more than PMv, could play a role in driving motor inhibition in JA tasks requiring reciprocal motor adaptations.

### Neural inhibition during JA tasks

The inhibitory indices we focused on have dissociable functions in motor control^53,54^ and, potentially, also in JA coordination. SICI reflects rapid intracortical M1 inhibition, mediated by GABAa receptors^55^ and is considered central to motor preparation^54,56^. Its modulation reflects preparation for a specific movement^57^, and in particular, the regulation of muscle synergies during complex hand actions^58^. At rest, cTBS on PMd^59^ or the strengthening of PMv-M1 connectivity by means of cc-PAS stimulation (cortico cortical paired associative stimulation) does not modulate sICI^60^. These results match with the present observation showing no TBS-induced modulation of either PMd or PMv. Consequently, although sICI was differentially modulated during JA and no-JA, it showed no sign of dependence on a specific premotor drive.

In fact, the premotor drive might reflect a more abstract level of motor representation^61^, one that may encompass adaptations across trials as opposed to within trials. In this sense, fast inhibition during JA might reflect the implementation of online fine adjustments, which may dissociate from the emergence, across trials, of a model of our partners’ behavior^7^. The lack of any sign of sICI modulation in relation to mutual learning of partners’ idiosyncrasy, might have made this index more robust to premotor perturbation.

In contrast, cSP duration reflects the activity of slow GABAb-mediated postsynaptic inhibition^62,63^. This index is associated with voluntary motor drive^64^ and is considered a marker of response selection^53^. In particular, cSP is considered a measure of corticospinal inhibition during voluntary movement, highlighting the optimization of movement planning to avoid erroneous or premature responses^65^. In agreement with our current findings, cSP appears to be modulated by the premotor cortex and more prominently by PMd^66,67^.

Importantly, cSP modulations have also been reported in studies investigating key components of JA, such as the integration/separation between motor representations of self and others. For instance, cSP is modulated for mismatching executed and observed actions^46^ (e.g. execute a hand opening action while observing a hand closing action). Interestingly, the integration/separation between motor representations of self and others is a necessary prerequisite for successful coordination in a JA settings^68–70^. Furthermore, modulations of cSP during JA explain motor adaptations across trials^7^, thus reflecting coregulatory mechanisms on a longer time-scale and possibly including trial-by-trial learning of partners’ individual motor styles^71^. Along these lines, it is worth mentioning that slow GABAergic activity is implicated in the regulation of LTP-like mechanisms that are essential to motor learning and plasticity^72,73^.

### Premotor modulations during JA tasks

Regarding the differential contribution provided by the lateral premotor cortices to JA control, both PMd and PMv coordinate visuomotor transformations to finely control reaching and grasping behaviors^20,74^. However, PMv and PMd are also characterized by important functional differences^75,76^. The PMd plays an important role in the control of arm movement^77^. This area, also via direct projections to spinal interneurons^78^ or via subcortical structures^79^, drives behavioral inhibition in monkeys^80,81^ and in humans^82^. These projections tune response preparation^83^ and action selection^84^, particularly in the context of visuomotor associations^83,85,86^. Action selection is indeed achieved by suppressing all competing movements that are prepared but not used^87,88^ and thus to promote the release of the most appropriate one^89–91^.

During JA, our brain not only represents what the partner is doing but also continuously reorganizes own action plans to improve JA coordination^3,92,93^. In fact, motor planning in this context must be integrated with predictions of others’ actions, anticipating the eventual need for corrections caused by violations of expectations^94,95^. Therefore, the PMd’s role in motor planning makes it a good candidate for regulating motor decisions, even during social coordination. This functional role is supported by a recent study that integrated a JA task (very similar to the present one) with a stop-signal task^24^. This study shows that cTBS interference on PMd induces modulation of the stop signal reaction time (SSRT), particularly when it is embedded in a JA condition.

On the other hand, the PMv contributes to the coordination of hand movements during grasping^96^ and neurons in the PMv encode target object features (such as shape and size) that are relevant to action goals^96,97^. The functional connection between the PMv and M1 implements a coordinate transformation between the extrinsic (visual-based) and intrinsic (muscle-based) movement spaces^74^ which is critical for controlling goal-directed actions^13,98^. Critically, PMv activity is modulated by the observation of other’s action^99,100^ and by contextual information (i.e., action goals) during movement execution^101^; thus, its contribution to the processing of partners’ motor signals during JA cannot be excluded. During JA, goal-related contextual information necessarily include the behavior of partners. Consistent with this fact, the PMv is recruited when monkeys have to withhold from executing an action because it is someone else’s turn, suggesting its role in monitoring their socio-interactive context^102^. Nevertheless, PMv seems to be focused on higher-order features of shared actions^30^, such as deciphering action goals^15^, rather than how to organize own movements towards JA goals.

Our JA task was specifically designed to tackle this latter component of JA. Here, participants are engaged in an interaction that elicits a refined but low-level motor co-adaptation of the spatiotemporal properties of reaching and haptic exchanges of forces. Importantly, we collected inhibitory indices during the reaching phase, locking our data acquisition to the activity of a muscle that is highly specific for the upcoming haptic interaction (i.e., OP). With this last choice, we have focused programmatically on a relatively low-level motor component on which JA goals, at least in our task, are necessarily built. These task and design choices might explain why the role of PMd emerged as more prominent than that of PMv. In fact, in JA, while PMv might contribute to action goal achievement by combining all contextual sensory information, PMd might integrate all the motor-relevant information required to generate the appropriate motor output^103^.

In conclusion, our results suggest that PMd plays a key role in the regulation of slow neural inhibition when motor co-adaptation between partners is required. This findings have fundamental relevance when considering that the balance between excitation and inhibition (E/I balance) has fundamental consequences for brain functioning^104^ and, when altered, it is believed to lead to a number of pathological changes^105^ such as the Autism Spectrum Disorder^106^ (ASD). While the E/I balance relies on GABAergic interneurons^107^, whose activity represents a significant part of brain metabolism^108^, inhibition at both cortical motor^29^ and spinal level^109^ is central to the fine regulation of individual motor production. The present results suggest that PMd could be a potential target for the treatment of disorders characterized by an altered E/I balance, which also affects the motor-interactive dimension, as in the case of some forms of autism^110,111^.

### Limitations of the study and future prospective

One limitation of our study is the absence of an appropriate assessment of the effect of cTBS, administered to the premotor cortex, on JA behavioral performance. In fact, although cTBS is likely to produce very weak effects on simple and overlearned motor tasks, here we could not test for such an interesting possibility. Unfortunately, the short duration of cTBS effects imposes a limit to the number of trials, notwithstanding the fact that there’s a limit to the number of trials before behavioral performance starts to change in unpredictable manners. In the present study, the aim was to replicate the dissociation between fast and slow inhibition in JA and describe which one was driven by premotor areas. For that reason, we optimized the design to collect those neurophysiological index at the expenses of the possibility to explore behavior in a quantitative manner. Future research would have to link JA coordination dynamics, different forms of inhibition and premotor influences to clear behavioral outcomes.

## Data Availability

The datasets recorded and analyzed in this study is available from the corresponding author on reasonable request.
